# A Multi-Dimensional Analysis of the Changing Role of Clinical and Community Pharmacists in Romanian Healthcare

**DOI:** 10.3390/healthcare14050624

**Published:** 2026-03-01

**Authors:** Alexandra Cristina Tocai (Moțoc), Felicia Dragan, Daria Marina Dragan, Andrei George Teodorescu, Cristina Oana Daciana Teodorescu, Camelia Florentina Ciobanu, Diana Uivarosan, Dana Carmen Zaha

**Affiliations:** 1 Department of Preclinical Disciplines, Faculty of Medicine and Pharmacy, University of Oradea, 410073 Oradea, Romania; diana.uivarosan@gmail.com (D.U.); danaczaha@gmail.com (D.C.Z.); 2 Department of Pharmacy, Faculty of Medicine and Pharmacy, University of Oradea, 410028 Oradea, Romania; 3 Department of Dental Medicine, Faculty of Medicine and Pharmacy, University of Oradea, 410073 Oradea, Romania; dariamarinadragan@gmail.com; 4 Department of Morphological Disciplines, Faculty of Medicine and Pharmacy, University of Oradea, 410073 Oradea, Romania; teodorescu_andrei_george@yahoo.com (A.G.T.); cami5ciobanu@yahoo.com (C.F.C.); 5 C.F.1 Witting Clinical Hospital, 010243 Bucharest, Romania; cristina.teodorescu2001@yahoo.com

**Keywords:** pharmaceutical care, clinical pharmacist, community pharmacist

## Abstract

Community and hospital pharmacists in Romania are valuable to healthcare, but their involvement in multidisciplinary teams is still not up to the mark when measured against international standards. A systematic literature review search was conducted using the PubMed, Embase, Web of Science, and Scopus databases, following the PRISMA 2020 guidelines, complemented by a bibliometric analysis with VOSviewer, to identify research trends and key contributors in the field. This review examines studies such as counseling effectiveness, clinical contribution, and professional obstacles faced by pharmacists in Romania published between 2014 and 2025 that mainly focus on community practice, integration into hospitals, and new areas such as pharmacogenetics and pharmacovigilance. The studies surveyed patients on how they evaluated counseling, tracked clinical pharmacists who adjusted drug doses through therapeutic monitoring, checked whether healthcare professionals followed safety warnings, and checked management systems within institutions. High costs and the removal of pharmacovigilance from compulsory university courses add to the obstacles. Romanian pharmacists already possess the knowledge or skills to raise treatment success and shield patients from unsafe self-medication through timely clinical advice. To use this capacity fully, the system must change, including health insurance covering pharmaceutical services, compulsory updating of course content, and official interdisciplinary protocols for this potential to be fully exploited.

## 1. Introduction

The World Health Organization (WHO) and the International Pharmaceutical Federation (IPF) now refer to the pharmacist as a caregiver, communicator, and leader who is part of the multidisciplinary healthcare team [1]. In the European Union, this change is reflected by structured medication utilization reviews, pharmacist-led immunization programs, and advanced clinical roles on hospital wards. On the other hand, in Romania, this change has a unique paradox. The Romanian healthcare system is chronically under-funded, and there are shortages of doctors in rural areas, but the country is well-stocked with pharmacies and has a very accessible network of pharmacists [2,3]. Even though pharmacists have greater accessibility, their role remains largely limited to dispensing medications. Although national legislation nominally recognizes clinical pharmacy, the practical integration of these professionals into hospital teams and primary care is often fragmented [4,5].

Romanian pharmacists offer full pharmaceutical care mainly in community pharmacies through various activities including dispensing of prescription and non-prescription medicines, patient counseling, medicine compounding, vaccine administration, testing health parameters, and carrying out health campaigns. All these activities are regulated by Law 266/2008, and the Pharmacies College supervises their implementation [6]. Apart from providing regular pharmacy services, Romanian pharmacists took part in various vaccination programs and health education campaigns during the COVID-19 pandemic, thus showcasing the extension of their public health role [7,8]. Pharmacist counselling typically follows a structured sequence: (a) assessment of the patient’s medication history and health status; (b) information provision on indication, dose, route, timing, and possible side effects and interactions; (c) verification of understanding through the teach-back method or open-ended questions; (d) adherence support (e.g., refill planning); and (e) documentation of the encounter in the patient record [7,8].

Academic programs in Romania have brought the curriculum up to EU standards, so that graduates have clinical skills in pharmacogenetics and therapeutic drug monitoring. However, these specialists, as soon as they start their professional careers, face institutional obstacles: lack of payment for cognitive services, excessive bureaucracy, and a legal framework oriented more towards organizational and supply chain safety mechanisms than towards clinical intervention [9,10]. Despite the fact that there is more and more data confirming the positive clinical impact of pharmacists in Romania in terms of patient safety and therapy optimization, their job descriptions are still somewhat informal, non-standardized, and their integration into the national health insurance system is very superficial [3]. This paper aims to fill the knowledge gap regarding how pharmacists’ responsibilities have shifted in practice by synthesizing a decade of research (2014–2025) to evaluate pharmaceutical healthcare from the perspective of both the clinical and community pharmacists in Romania and to outline its development path.

## 2. Materials and Methods

### 2.1. Study Design

This study was realized as a systematic review, and it followed PRISMA 2020 guidelines. A structured search strategy was implemented in PubMed, Embase, Web of Science, and Scopus databases. These keywords and Boolean combinations were used: “clinical pharmacist”, “community pharmacist”, “pharmaceutical care”, “medication therapy management”, and “Romania”. The search covered publications indexed between 1 January 2014 and 1 January 2026. Studies published between 2014 and 2025 that met the eligibility criteria were included in the final review.

### 2.2. Eligibility Criteria

Studies were selected according to predefined inclusion and exclusion criteria. Inclusion criteria were primarily based on studies conducted in Romania, with papers published between 2014 and 2025, written in Romanian or English, and studies involving pharmacists or aspects of pharmaceutical practice, whether from community or hospital pharmacies. Observational studies, cross-sectional surveys, interventional studies, descriptive analyses, policy evaluations, and pharmaceutical practice-based research were considered.

The exclusion criteria considered were conference abstracts without full availability or editorials, opinion articles, and articles that do not have a direct link to pharmaceutical practice or to the specific activities carried out by pharmacists.

### 2.3. Study Selection Process

**Figure 1:**
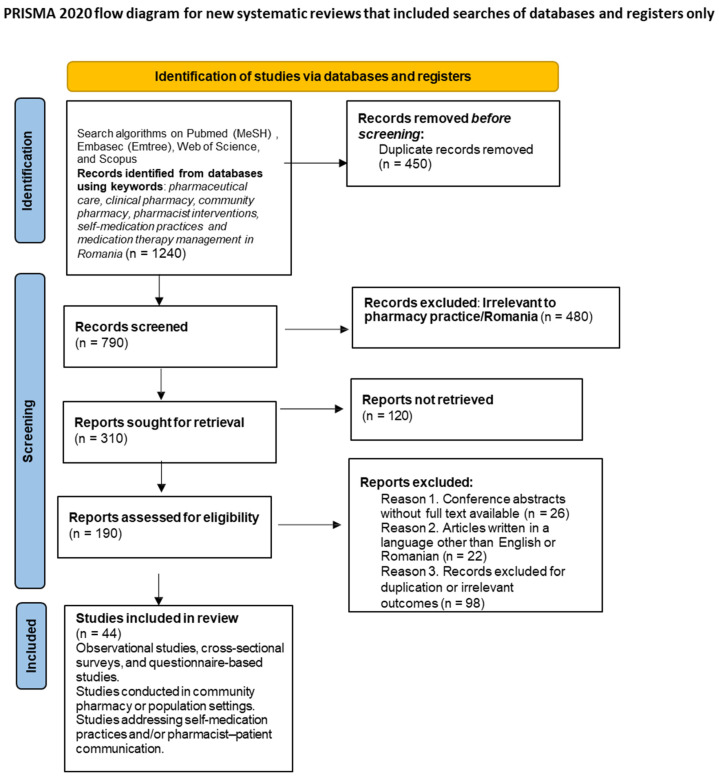
PRISMA flowchart illustrating the identification, screening, eligibility, and inclusion of studies on clinical and community pharmacists in Romania.

The study selection process is summarized in a PRISMA flowchart [11] (Figure 1), which complements the bibliometric analysis.

A total of 1240 records were identified by searching different databases. After removing 450 duplicate records, 790 titles and abstracts remained and were further analyzed for their relevance to the subject of the analysis. Of these, 480 records were excluded because they did not meet the inclusion criteria (not related to pharmaceutical practice or not conducted in Romania). Full texts of 310 reports were requested for retrieval, of which 120 were not available. The eligibility of 190 full-text articles was checked, and after applying the exclusion criteria (conference abstracts without full text, articles in other languages, irrelevant scope, or lack of pharmacist involvement), 44 studies were finally included in the analysis. All database-specific search strategies are provided in Supplementary Table S1 to ensure transparency and reproducibility.

The 44 studies covered in the systematic review are the References [12,13,14,15,16,17,18,19,20,21,22,23,24,25,26,27,28,29,30,31,32,33,34,35,36,37,38,39,40,41,42,43,44]. References [1,6,10,11,39] were only cited to provide the international contextual background and have not been included in the systematic review dataset reflected in the PRISMA flow diagram.

### 2.4. Bibliometric Analysis

**Figure 2:**
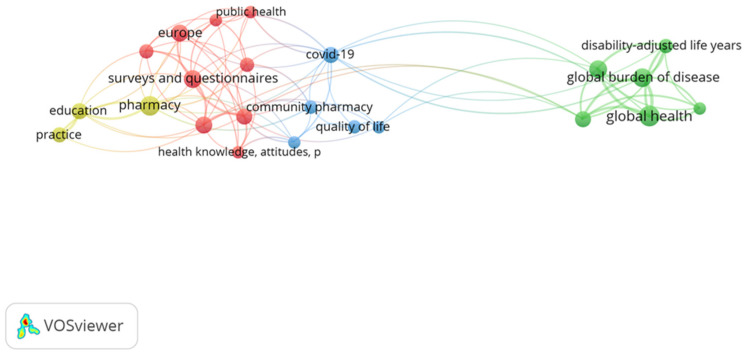
Knowledge mapping of high-frequency keywords in pharmacist-led research (2014–2025).

A bibliometric analysis of author keywords was conducted using VOSviewer (version 1.6.20; Leiden University, Leiden, The Netherlands) (Figure 2). A total of 609 keywords were identified from the included publications. To enhance interpretability, a minimum occurrence threshold of five was applied, resulting in 40 keywords being included in the final co-occurrence network. The visualization map represents keywords as nodes, with node size proportional to their frequency of occurrence. Links between nodes indicate co-occurrence relationships, with link strength reflecting the frequency of joint appearances. Clustering was performed automatically by VOSviewer to identify major thematic areas within the literature. The keywords in the list are categorized into 4 clusters, reflecting the central study regions of the study. Cluster 1, presented as the Red Cluster, underpins the community pharmacy. This is a dominant topic specializing in public health, Europe, and using surveys and questionnaires. It bridges the gap between community pharmacy settings and broader health knowledge and attitudes. Cluster 2, or the Green Cluster, is about discussion on reimbursement and workforce distribution. This distinct thematic vicinity focuses on macroeconomic health metrics, which include global health, the worldwide burden of disease, and disability-adjusted life years. Cluster 3, or the Blue Cluster, refers to the expansion of services caused by the pandemic, inspecting its effect on community pharmacy and the resulting quality of life for patients. Cluster 4, or the Yellow Cluster, highlights the foundational aspects of the field, in particular, it specifically focuses on pharmacy education and the evolution of professional practice development.

The thematic clusters identified through the bibliometric analysis informed the structural organization of the review. The Red Cluster, or public health, surveys, and community pharmacy practice, lines up with Section 3, which examines community pharmacists’ counseling and patient perception. The Green Cluster, which covers global health indicators and the burden of disease, underpins Section 4 and Section 9, which focus on clinical pharmacist impact and patient safety. Then, the Blue Cluster, or COVID-19 and quality of life, relates to Section 5 and Section 6, addressing advanced roles and crisis-driven professional expansion. The yellow cluster is all about education and professional development in pharmacy, and aligns with Section 6, which tackles education, workforce trends, and professional identity. This whole bibliometric mapping not only visualizes research trends but also guides the thematic synthesis presented in Section 3, Section 4, Section 5, Section 6, Section 7, Section 8, Section 9 and Section 10.

## 3. Community Pharmacist Counseling and Patient Perception

As identified in the bibliometric analysis, community pharmacy practice and public health-oriented survey research represent a dominant thematic group (Cluster 1 or the Red Cluster), forming the foundation of the following section.

Multiple surveys carried out in Romanian community pharmacies show that pharmacists provide medication counseling every day. The same studies keep finding blind spots; patients rarely hear how the drug might change blood test results, when it expires, or how to bring back leftover tablets, especially psychotropics [9].

Nevertheless, identifying qualitative gaps was a continuous feature of the studies. Concordance studies also revealed that there were differences between the pharmacists’ self-reported counseling and the patients’ perceptions, especially in the case of safety-related information such as adverse drug reactions, where the level of agreement was very low. Community pharmacists are also at the forefront of handling minor ailments and guiding self-medication. According to surveys, analgesics and gastrointestinal drugs are in daily demand, while antibiotic self-medication for serious illnesses is at an alarming level. Experienced pharmacists were less likely to promote inappropriate self-medication, thus professional judgment and communication skills are of great importance [12,13]. All relevant studies are summarized in Table 1.

## 4. Clinical Pharmacist Impact on Patient Safety

Across hospital and clinical research settings, it has been demonstrated that clinical pharmacists’ efforts in medication management greatly improve patient safety and health outcomes [17]. All studies related to the clinical pharmacist are summarized in Table 2.

Within ICU, geriatric, and infectious-ailment settings, Romanian records reliably suggest that clinical pharmacists considerably enhance patient well-being. Their movements start from therapeutic drug oversight and renal-dosage tuning to systematic medicine evaluation using STOPP/START guidelines, and oversight of limited antibiotics addresses principal origins of avoidable problems like kidney damage, prescribing gaps, and antibiotic overuse.

Although individual studies demonstrate safety gains, the clinical pharmacy services in Romanian healthcare remain fragmented across institutions because they are unpaid, lack systematic EHR support, and are not embedded in national policies.

## 5. Pharmacovigilance, Safety, and Advanced Roles

Several studies highlight the untapped potential of pharmacists to play a major role in pharmacovigilance, contrasting it with current legislative and educational deficiencies [22]. Pharmacists indicated that safety communications had changed their patient counseling practices the most. Comparative studies between the Romanian legal framework and European standards of Good Pharmaceutical Practice have shown that pharmacovigilance education should not be considered optional and that the pharmacist’s role is mainly to inform patients and report adverse reactions [23].

Highly professional functions, including systematic tracking and subsequent procedures, are being developed. This section suggests that, despite the fact that Romanian pharmacists are legally considered contributors to pharmacovigilance, the lack of mandatory training, the lack of organizational alignment, and the non-existence of standardized safety services limit them to providing only slightly improved affected person confidentiality, and public health interventions are strongly contradicted because it has been demonstrated that they may each be possible and essential [24]. A summary of studies about pharmacovigilance is provided in Table 3.

This series of evidence suggests that Romanian pharmacists are acknowledged but underused pharmacovigilance contributors. While state-of-the-art capabilities in risk reduction, dosage refinement, immunization, and emergency response are demonstrably effective, their implementation is limited by legal, academic, and structural obstacles. Resolving those deficiencies is essential to completely leverage pharmacists’ capability in improving affected patient health and public health.

## 6. Education, Professional Identity, and Workforce Issues

Studies exploring the perceptions of healthcare professionals have established a substantial reputation for the significance of interdisciplinary collaboration. While nearly all respondents stated its value, poor communication was often reported as having a negative impact on affected patient care. Over half of healthcare professionals believed that integrating clinical pharmacists into healthcare groups might extensively enhance healing consequences of early detection of interactions and optimization of treatment [30].

Ethical issues have additionally been raised, especially around the affected person’s confidentiality, highlighting systemic issues that extend beyond pharmaceutical practice. During the COVID-19 pandemic, pharmacists’ extended roles in testing, vaccination support, and public health education have similarly established their adaptability and clinical application while regulatory boundaries have been temporarily relaxed [29]. A summary of studies on the professional development of pharmacists in Romania is provided in Table 4.

This section underscores a structural gap amongst education, professional status, and workforce balance in Romanian pharmacists. While pharmacists welcome superior features and EU-conforming educational structures are present, minimal investment for healthcare, constraining statutes, restricted clinical independence, and insufficient post-graduate possibilities impede professional advancement, satisfaction, and ultimately constrain the development of pharmacy practice. Entry-level pharmacy education in Romania is at a level with European standards and gives students a basic knowledge of clinical practice. However, to be specialists in pharmacogenetics, antimicrobial stewardship, therapeutic drug monitoring, and management of chronic diseases, pharmacists need structured postgraduate programs. Just as it is the case in other health professions, greater clinical authority of pharmacists should be backed by formal certification, supervised clinical training, and continuous professional development (CPD). A few extended services form the basic level of competencies (e.g., medication counselling), while others require the need for formal education and credentialing for frontline professionals in order to ensure patient safety and professional accountability.

## 7. Interdisciplinary Collaboration and Policy Barriers

Based on the national survey, it is evident that, in general, pharmacists in Romania have favorable views towards advanced practices such as pharmacogenetics, medication therapy management, and patient-centered care. On the other hand, an in-depth analysis of the pharmacy workforce reveals a scenario in which workers show a lack of satisfaction with the law, salaries, and budget limitations, considering how much they had to study during their education [33]. Furthermore, a comparative assessment of Good Pharmaceutical Practice requirements amongst countries additionally indicates that Romanian guidelines are at a lower level than worldwide ones concerning acknowledgment and charge of pharmaceutical cognitive services [34]. A summary of studies about pharmacy practice policy is provided in Table 5.

This section illustrates that in the Romanian healthcare system, interdisciplinary collaboration is not always limited by specialist approval, but by political, legal, and organizational impediments. Although findings and practitioner perspectives certainly prefer the inclusion of pharmacists in various groups, restrictive rules, absence of fees for duties, and structural sluggishness limit their active involvement in treatment choices. International and local examples reveal that after policy setups enable partnership, pharmacists considerably increase safety, efficiency, and results for patients.

Across all themes, the proof reliably indicates that Romanian pharmacists presently offer enormous resources to patient aid and drug security, mainly in which clinical pharmacy services are established. However, those contributions stay fragmented, inconsistently recognized, and insufficiently supported by legislation, fee systems, and structured training pathways.

## 8. Community Pharmacy Practice in an International Context

Internationally, community pharmacists are often considered providers of only recently structured services, such as Medication Utilization Reviews in the United Kingdom, Medication Initiation Services in Denmark, Medication Therapy Management Programs in the United States, and Medication Review Services, which are reimbursed in several EU health systems [39,40]. These interventions are standardized, protocol-based, documented, and financially remunerated, with a wealth of evidence supporting improvements in medication adherence, reduction in medication-related problems, and patient safety. Comparatively, community pharmacists in Romania offer detailed medication counseling, as word-of-mouth testimony to advise on dosage, administration, and interactions is extremely high. In fact, these rates are often higher than those of international benchmarks. However, contrary to structured and reimbursed international pharmaceutical services, medication counselling in Romania is often informal, variably documented, and not financially compensated [18,34]. Pharmaceutical counselling, in contrast to psychological counselling, is drug-focused and clinically directed with the purpose of enabling patients to make informed decisions and manage their conditions safely [18].

Although pharmacists frequently provide detailed medicine-related advice, the absence of standardized documentation systems and performance indicators makes it difficult to measure clinical impact or integrate these activities into national healthcare quality metrics [13].

Among the recurring themes of Romanian research on community pharmacy is the finding that there is a significant gap between the pharmacists’ self-reported counselling behavior and the patients’ understanding of it, especially regarding adverse drug reactions. This phenomenon has also been described globally, but the data from Romania reveal a greater disparity, which is probably due to the limited professional training in patient-centered communication and the lack of standardized counselling frameworks [18,27]. International frameworks increasingly revolve around concepts such as sharing decision-making, risk communication, and the use of structured counselling checklist techniques [41], which could be used to tackle the gaps that have been identified in the practice of community pharmacy in Romania.

Furthermore, the involvement of Romanian community pharmacists in self-medication management is, on the one hand, the source of increasing opportunities and, on the other hand, the source of risks from an international point of view. Just like the rest of the world, patients rely on pharmacists as their first-line healthcare providers when ailments are minor [13,24]. These results point to a pressing need for clearer professional authority, focused public communication, and enhanced integration of community pharmacists in primary care pathways in Romania.

## 9. Clinical Pharmacy and Patient Safety: Romania Versus International Evidence

Worldwide evidence demonstrates that clinical pharmacist-led initiatives have been shown to markedly enhance patient safety, therapeutic outcomes, and health system efficiency [42]. In nations with a well-developed clinical pharmacy sector, pharmacists are essential members of multidisciplinary teams and perform functions such as therapeutic drug monitoring, antimicrobial stewardship, medication reconciliation, and organizing medication reviews. Such interventions lead to fewer adverse drug reactions, medication errors, shorter hospital stays, and lower healthcare expenditure. The observations of Romanian hospitals are remarkably in agreement with the international results; however, the number and coverage of studies are limited. Pharmacists in Romanian ICUs were able to identify a large extent of initial vancomycin dose errors, which is in line with what has been reported worldwide [19]. This implies that Romanian pharmacists, when given the clinical authority to intervene, can deliver safety outcomes comparable to those reported in advanced clinical pharmacy systems on the global scene; it has been proven that the regular participation of clinical pharmacists in stewardship programs contributes to lessening the extent of inappropriate antibiotic use and therefore fights antimicrobial resistance. In fact, Romanian data confirm this approach, revealing that pharmacist engagement could greatly enhance the quality of prescribing; however, such involvement is still implemented sporadically, as it depends on local initiative rather than being driven by national policy.

Medication review investigations of elderly patients in Romania have also helped to highlight the agreement between local and global evidence. Employing validated instruments such as STOPP/START criteria, clinical pharmacists found a wide range of drug-related problems, including inappropriate drug use and prescription of unnecessary drugs in some cases [20]. Such discoveries go hand in hand with the international literature, which indicates that pharmacist-led medication reviews bring about reductions in morbidity, hospital admissions, and help older people live better lives. Yet, whereas in many European nations medication review is part of the healthcare system and funded [43], Romanian measures continue to be fragmentary and experimental. From a policy standpoint, the rollout of expanded pharmacist services calls for a sound use of resources. Domestic and worldwide examples, including integrated health systems like Kaiser Permanente (USA), show that pharmacists’ interventions in chronic disease management and medication review lead to a decline in hospital admissions, emergency visits, and long-term complications; thus, cost savings can be used to pay for services [42]. Economic evaluations of healthcare are frequently the main instrument to convince government and insurance authorities to pay for cognitive pharmaceutical services. Lack of credible Romanian cost-effectiveness studies is a major obstacle, a threat, and a challenge, and at the same time, an opportunity in the field of national pharmacy practice reform.

In general, comparing Romanian with international data points to an identical pattern: the clinical effects of pharmacist-led interventions in Romania are in line with the ones indicated internationally; however, their execution is devoid of sustainability, standardization, and policy backing. This difference highlights that the main constraint of clinical pharmacy in Romania is not skills and professionalism but rather the lack of structural integration, legal recognition, and reimbursement systems that would grant pharmacists the ability to fully engage in ensuring patient safety nationwide.

## 10. Pharmacovigilance and Advanced Roles in a European Perspective

Pharmacists are becoming increasingly identified within Europe as integral professionals within pharmacovigilance, and their role is no longer confined to the passive reporting of drug reactions but also includes the areas of active surveillance, follow-up, risk minimization, and safety educational activities. Within some European states, the skills of pharmacovigilance are incorporated into the postgraduate and undergraduate curricula and are encouraged by strict regulatory and development activities. Evidence from Romania suggests that a good level of knowledge and awareness of important safety communications, like those regarding adverse reactions with fluoroquinolones, identified in Direct Healthcare Professional Communications, has been observed in pharmacists [27,44]. However, their level of knowledge and concordance with their behaviors seem moderate, especially when considering their engagement in a later stage after safety communications by regulatory bodies. This has been consistent with findings that single safety communications cannot provide constant pharmacovigilance to ensure effective engagement with regular training. One important departure from best practices in Europe regards the regulatory and educational environment for pharmacovigilance in Romania. While these best practices include mandatory training for pharmacovigilance and well-defined professional roles, such regulatory restrictions in Romania have, until recently, confined professional roles for pharmacists merely to counseling patients and submitting spontaneous reports. The optional aspect of pharmacovigilance educational training in Romania also limits the ability of pharmacists for advanced safety work, such as monitoring patients on high-risk therapies [44]. New fields of highly developed practice for pharmacists, such as pharmacogenetics and personalized medicine, also demonstrate the discrepancy between the Romanian and European implementation levels. The enthusiasm and attitude toward pharmacogenetic practice, shared by pharmacists in Romania, are identical to those found in Europe. However, reduced confidence, lack of education, cost associated with the test, and no system to obtain reimbursement stand between pharmacogenetic practice and its implementation in Europe. Meanwhile, pharmacogenetic practice has already found its implementation in the healthcare system of Europe [31].

In general, when examined from a European perspective, Romanian pharmacists are ready and motivated to engage with expanded roles of pharmacovigilance and advanced clinical practice. For the Romanian system to conform to European standards, there would need to be more than legislative changes invested if their potential in ensuring pharmaceutical safety is to be fully realized.

## 11. Limitations

Taken together, the evidence reviewed highlights a continual gap between pharmacists’ established scientific ability and their formal position inside the Romanian healthcare system. These barriers are, in the main, structural and regulatory rather than professional. Table 6 summarizes the current state of pharmacy practice in Romania, the corresponding required future state, and the important movements required to permit the transition towards patient-centered pharmaceutical care.

## 12. Conclusions

The data collected from 2014 to 2025 affirms that the Romanian pharmacist represents a vastly underused resource within the healthcare system. The outcomes show a professional organization that is addressing the scientific understanding by significantly lowering prescription mistakes, overseeing long-term illnesses, and enhancing affected person confidentiality, but it is restricted by an out-of-date operational structure. While local pharmacists provide common guidance, there is a remarkable divide among pharmacists and patients regarding vital protection information, including adverse drug events. In clinical settings, pharmacist-led interventions, especially in vancomycin TDM and antimicrobial oversight, have proven successful in lessening kidney harm and prescribing faults, matching worldwide success rates. The elimination of pharmacovigilance from obligatory coursework and the absence of payment for professional services (immunization, medicinal drug reviews) act as the most important deterrents to expert development. In conclusion, understanding the entire functionality of the Romanian pharmacist is not simply a professional goal; it is a public health necessity. Defining these capabilities officially will result in a more resilient, secure, and less expensive healthcare structure for the Romanian population. Taken together, the constraints affecting Romanian pharmacy practice are not associated with professional competence; rather, the constraints are associated with structural underutilization, regulatory inertia, and inadequate system-level integration.

## Figures and Tables

**Table 1:** An overview of the studies based on community pharmacists and patient perception.

Methodology and Scope	Main Focus	Principal Outcomes	Pharmacist–Patient Communication Conclusions	References
Descriptive multicenter survey; 300 pharmacists	Pharmacists’ perspectives on self-medication	- Self-medication is common, 19.1% implicated antibiotics. - Experienced pharmacists are much less likely to inspire self-medication.	Shows how professional experience shapes counseling rigor and gatekeeping behavior.	[13]
Cross-sectional survey; 506 pharmacists	Patient-targeted communication skills	- Pharmacists self-rate communication as good; restrained shared decision-making (6.5%). - Lack of training in the “patient-centered communication” concept.	Reveals attitudinal boundaries to enhancing high-satisfaction counseling of affected patients.	[14]
Cross-sectional paired questionnaires; 2047–2378 encounters, 520 pharmacies	Agreement between pharmacists’ and patients’ perceptions	- Highest agreement (PABAK ≥ 0.80) was on counselling regarding drug administration route, dosage, and time. - The biggest difference (PABAK = 0.01) was on adverse drug effects (ADRs), where only 44.8% of patients reported being counselled, whereas 93.1% of pharmacists said they provided it.	Highlights the perception gap and overestimation of counseling quality by pharmacists.	[15]
Observational study; 289 patients; pharmacist-administered questionnaire	Adherence, QoL, non-pharmacologic factors	- Adherence is strongly encouraged through health education, disease knowledge, BP self-monitoring, and consultation frequency. - Pharmacist performed a key position in data collection and patient education	Demonstrates the indirect impact of pharmacist counseling on adherence and QoL.	[16]
Legislative and practice-based analysis; symptom occurrence data	Counseling and dispensing under national legislation	- Pharmacists manage moderate GI symptoms with OTCs and refer severe cases. - Strict adherence to GPP and ANMDM rules; PPIs and H2RAs are prescription-only. - Personalized counseling reduces self-medication abuse.	Illustrates counseling as a regulatory and patient safety tool.	[12]
Cross-sectional comparative survey; robust regression analysis; 343 patients	Determinants of patient satisfaction with community pharmacy services versus trust in information received	- Expected satisfaction through pharmacist attitude (β = 0.631), reduced waiting time, availability and cost of medications, provision of precautionary information, and patient condition (non-chronic patients have more confidence).	- Demonstrates that satisfaction and trust are driven by different communication behaviors: - a polite attitude enhances service satisfaction, while attentive, information-rich counseling builds trust and helps pharmaceutical care adoption.	[17]
Prospective, non-interventional survey; 2453 patients, 520 pharmacies	Patient-reported pharmacist counseling	- >90% acquired recommendation on administration, dosage, and timing; - >80% on contraindications, interactions, and ADRs. - Low counseling on unused psychotropic drug return (38%) and lab test interference (47.6%).	Demonstrates high perceived counseling quality, but identifies gaps in safety-critical and public-health-oriented counseling.	[18]

ADRs—adverse drug reactions; ANMDM—The Romanian National Agency for Medicines and Medical Devices; BP—Blood Pressure; GI—Gastrointestinal; H2RAs—histamine type 2 receptor antagonists; QoL—quality of life; PABAK—prevalence-adjusted biased adjusted kappa; and PPIs—proton pump inhibitors.

**Table 2:** Evidence on the impact of clinical pharmacists on patient safety in Romanian hospital practice.

Methodology and Scope	Patient Safety Focus	Key Clinical Pharmacist Contributions	Main Outcomes and Conclusions	References
Retrospective, single-center pilot study; ICU patients (January 2023–December 2024 versus August 2021–December 2022)	Avoiding nephrotoxicity and maximizing antibiotic treatment	- Pharmacist-guided vancomycin TDM; PK/PD analysis; - Dose individualization; - Kidney function observation	- 62% of preliminary doses were insufficient and needed adjustment; - Medication monitoring led by pharmacists decreased kidney impairment incidence by 34%. - Supports integration of clinical pharmacists and standardized TDM in ICU treatment.	[19]
Cross-sectional prescription examination; ~1498 chronic prescriptions; community and primary care	Medication safety in seniors; prevention of morbidity from prescribing deficits	- Clinical pharmacist-led medication evaluation; - Application and contextualization of STOPP/START v.2 criteria; - Polypharmacy appraisal	- Widespread PPOs (49.2%) versus PIMs (18.6%); - Common omissions included statins and beta-blockers; - Authors suggest establishing pharmacist-led medication evaluations and insurance via national insurance.	[20]
Retrospective assessment; 664 limited antibiotic requests; academic hospital	Rational antibiotic use and resistance prevention	- Clinical pharmacist involvement in prescription authorization; - Dose and period correction; TDM; - Kidney dose adjustment; step-down support	- 42.9% of prescriptions unsuitable; - Misuse is highest for vancomycin and carbapenems; - Clinical pharmacist input and formularies are recognized as crucial to lower unsuitable usage, costs, and resistance.	[21]

PD—pharmacodynamics; PIMs—potentially inappropriate medications; PK—pharmacokinetics; PPOs—Potential Prescribing Omissions; STOPP/START—Screening Tool of Older Persons’ Prescriptions/Screening Tool to Alert to Right Treatment; and TDM—therapeutic drug monitoring.

**Table 3:** Pharmacovigilance, safety, and advanced roles of pharmacists in Romania.

Methodology and Scope	Pharmacovigilance/Advanced Role Examined	Main Results	Implications for Expanded Pharmacist Roles	References
Cross-sectional survey; 267 community pharmacists	Risk minimization and regulatory compliance	- Low awareness and implementation of PPP; - Only 38.6% advised on teratogenic risk, and 15.2% continuously used educational materials.	- Reveals critical gaps in pharmacovigilance for high-risk drugs; - Involves the need for training, availability of materials, dedicated counseling time, and remuneration.	[25]
Cross-sectional patient survey; 150 participants	Preventive care and vaccination delivery	- 68% favorable attitude toward pharmacy vaccination; - Only 36% aware of service availability; - ~34% assured in pharmacist-administered vaccination.	- Shows the untapped ability of pharmacists in immunization; - Emphasizes the need for structured patient education and communication strategies.	[26]
Policy and regulatory evaluation versus Pharmaceutical Group of the European Union Best Practice	Community pharmacist participation in pharmacovigilance	- Romanian legislation limits pharmacists to reporting adverse reactions and patient information; - Pharmacovigilance education became optional in 2021; - Lack of EHR access and insurance integration.	- Highlights major gap between EU expectations and national practice; - Calls for mandatory training, systemic integration, and formal recognition of pharmacists as active pharmacovigilance agents.	[27]
Descriptive evaluation of EV database reports	Medication safety and dose optimization	- Underdosing represented ~52% of dosing mistakes; - Fatal consequences noted particularly with overdosing; - Rivaroxaban is most frequently involved.	- Supports collaboration between pharmacists and physicians; - Pharmacist-led medication review to reduce dosing errors and life-threatening adverse consequences.	[28]
Narrative overview; EU and global perspective	Crisis-driven growth of pharmaceutical care	- Pharmacists supported vaccination, testing, telepharmacy, misinformation control, and emergency supply chains; - Telepharmacy use increased markedly.	- Demonstrates the feasibility of superior roles in emergencies; - Underscores the need for standardization and infrastructure to sustain these services post-pandemic.	[29]

EHR—Electronic Health Record; EV—EudraVigilance; PPP—pregnancy prevention program.

**Table 4:** Education, professional identity, and workforce challenges in Romanian pharmacy practice.

Methodology and Scope	Strategic Priority	Main Results	Implications for Education, Identity, and Workforce	References
National cross-sectional survey; 1058 pharmacists	Advanced aptitudes and personalized medicine	- Moderate pharmacogenetics (PGx) knowledge; - Effective attitudes but low readiness (only 14.7% confident to talk about PGx); - Younger pharmacists and pharmacists from hospitals/universities performed better; - Multiple systemic boundaries beyond cost identified.	- Reveals a mismatch between emerging professional roles and current education; - Highlights the need for curriculum reform, postgraduate training, compensation mechanisms, and clearer clinical pathways.	[31]
National policy and education analysis	Workforce distribution and education harmonization	- EU-aligned education structure, but persistent urban–rural imbalance and significant graduate mind drain; - Low healthcare spending limits salaries, infrastructure, and specialization development.	Demonstrates that instructional harmonization alone cannot sustain the workforce without adequate funding, incentives, and professional opportunities.	[32]
Cross-sectional study; 78 hospital pharmacists	Professional satisfaction and legislative impact	- High satisfaction with interpersonal relations; - Strong dissatisfaction with pay, promotion, budget, and legislation. - Limited clinical involvement reduces professional autonomy and advancement.	Shows how restrictive legislation undermines professional identity, professional progression, and motivation despite a positive workplace culture.	[17]

PGx—Pharmacogenetics.

**Table 5:** Interdisciplinary collaboration and policy barriers affecting pharmacy practice in Romania.

Study Design	Policy Focus	Key Findings	Policies and Practices in Interdisciplinary Practices	References
Cross-sectional perception survey; 160 Romanian healthcare professionals	Perceived value of clinical pharmacists	- >50% of clinicians believe pharmacists might enhance care quality; - Poor communication pronounced through 65.6%; - Pharmacists are viewed as underused despite evidence of benefit.	Reveals a mismatch between diagnosed capacity and actual integration of pharmacists into care teams.	[35]
Policy and practice analysis	Service recognition and remuneration	- Romanian pharmacists provide extensive counseling without remuneration; - In contrast to the UK/Denmark, services are not formally funded or standardized.	Highlights policy failure to apprehend and incentivize collaborative, patient-centered pharmaceutical services.	[34]
Comparative legal and policy analysis	Regulatory scope of pharmacy practice	- Romanian good pharmacy practice focuses on organizational safety; - Lacks medicine remedy control, vaccination, and clinical service standards present in FIP/WHO guidelines.	Identifies regulatory misalignment as a key barrier to interdisciplinary and clinical pharmacist roles.	[36]
Retrospective organizational analysis; Romanian hospital	Institutional management and internal collaboration	- IMCS, led by the chief pharmacist, enhanced communication, inventory control, waste management, and antibiotic use; - Model scaled hospital-wide.	Shows how organizational policy tools can strengthen pharmacy integration and operational collaboration even without expanded clinical authority.	[37]
Descriptive case-based study; Canada versus Romania	Systematic pharmaceutical care approach for elderly patients	- In Canada, pharmacist interventions were carried out inside a multidisciplinary team; - In Romania, guidelines remained hypothetical due to a lack of collaboration and limited pharmacist access to patients.	- Illustrates stark assessment among functional interdisciplinary models and Romanian practice; - Highlights systemic barriers preventing pharmacists’ active therapeutic role.	[38]

FIP/WHO—International Pharmaceutical Federation/World Health Organization; IMCS—Internal Managerial Control System.

**Table 6:** Current limitations of pharmacists in Romania.

Current State (2014–2025)	Required Future State	Action Required
Product-focused	Service-focused	Change Law 266/2008 to include Clinical Services
Voluntary ADR reporting	Active pharmacovigilance	Mandatory EHR integration for pharmacists
Informal counseling	Standardized protocols	National implementation of MUR
Limited clinical authority	Collaborative practice	Legal framework for pharmacist–physician collaboration
Fragmented clinical roles	System-integrated practice	National standards for clinical pharmacy positions
Variable training uptake	Mandatory continuous education	Compulsory CPD in clinical pharmacy and pharmacovigilance

ADR—adverse drug reaction; CPD—Continuing Professional Development; and MUR—Medication Use Review.

## Data Availability

No new data were created or analyzed in this study. All data were extracted from publicly available published studies.

## Supplementary Materials

The following supporting information can be downloaded at: https://www.mdpi.com/article/10.3390/healthcare14050624/s1, Table S1: Detailed Search Strategy and Database Information.

## Abbreviations

The following abbreviations are used in this manuscript:

ADRs	Adverse Drug Reactions
ANMDM	The Romanian National Agency for Medicines and Medical Devices
BP	Blood Pressure
CPD	Continuing Professional Development
EHR	Electronic Health Record
EV	EudraVigilance
FIP/WHO	International Pharmaceutical Federation/World Health Organization
GI	Gastrointestinal
H2RAs	Histamine Type 2 Receptor Antagonists
IMCS	Internal Managerial Control System
MUR	Medication Use Review
PD	Pharmacodynamics
PGx	Pharmacogenetics
PIMs	Potentially Inappropriate Medications
PK	Pharmacokinetics
PPIs	Proton Pump Inhibitors
PPOs	Potential Prescribing Omissions
PPP	Pregnancy Prevention Program
QoL	Quality of Life
STOPP/START	Screening Tool of Older Persons’ Prescriptions/Screening Tool to Alert to Right Treatment
TDM	Therapeutic Drug Monitoring
