# Supplementary material for: A Multi-Dimensional Analysis of the Changing Role of Clinical and Community Pharmacists in Romanian Healthcare
Source: Healthcare (Basel). 2026 Mar 1;14(5):624. doi: 10.3390/healthcare14050624 (PMC12984617; doi:10.3390/healthcare14050624)
Supplement: Supplementary file 1 [file healthcare-14-00624-s001.zip › healthcare-4146921-supplementary.pdf]

Table S1: Detailed Search Strategy and Database Information

Search period: 1 January 2014 – 1 January 2026

Date of final search: 1 January 2026. Studies published between 2014 and 2025 that met the eligibility criteria were included in the final review.

Databases searched: PubMed (MEDLINE), EMBASE, Web of Science Core Collection, Scopus

Concepts combined:

1. Pharmacist role / pharmaceutical services
2. Romania

**Database**	**Search strategy**
Search platform: PubMed<br>Controlled vocabulary: MeSH<br>Fields searched: MeSH Terms, Title/Abstract<br>Date searched: 1 January 2026<br>Filters applied:<br>• Publication date: 2014–2026<br>• Humans	1. "Pharmaceutical Services"[MeSH]<br>2. "Clinical Pharmacy"[MeSH]<br>3. "Community Pharmacy Services"[MeSH]<br>4. "Medication Therapy Management"[MeSH]<br>5. "Pharmacovigilance"[MeSH]<br>6. "Therapeutic Drug Monitoring"[MeSH]<br>7. "Clinical pharmacist"[Title/Abstract]<br>8. "Community pharmacist"[Title/Abstract]<br>9. "Pharmaceutical care"[Title/Abstract]<br>10. "Medication therapy management"[Title/Abstract]<br>11. "Medication review"[Title/Abstract]<br>12. 1 or 2 or 3 or 4 or 5 or 6 or 7 or 8 or 9 or 10 or 11<br>13. "Romania"[MeSH Terms]<br>14. "Romania"[Title/Abstract]<br>15. 13 or 14<br>16. 12 and 15<br>17. ("2014/01/01"[Date - Publication] : "2026/01/15"[Date - Publication])<br>18. 16 and 17
Search platform: Elsevier EMBASE<br>Controlled vocabulary: Emtree<br>Fields searched: Title, Abstract, Keywords<br>Date searched: 1 January 2026<br>Limits applied:<br>• Publication years: 2014–2026<br>• Article type: research articles	1. 'Clinical pharmacy'/exp<br>2. 'Community pharmacy'/exp<br>3. 'Pharmaceutical care'/exp<br>4. 'Medication therapy management'/exp<br>5. 'Pharmacovigilance'/exp<br>6. 'Therapeutic drug monitoring'/exp<br>7. 'Medication review'/exp<br>8. 'Clinical pharmacist':ti,ab,kw

	9. 'Community pharmacist':ti,ab,kw<br>10. 'Pharmaceutical care':ti,ab,kw<br>11. 1 or 2 or 3 or 4 or 5 or 6 or 7 or 8 or 9 or 10<br>12. 'Romania'/exp<br>13. Romania:ti,ab,kw<br>14. 12 or 13<br>15. 11 and 14<br>16. 15 and [2014-2026]/py<br>17. limit 16 to article
Search platform: Clarivate Web of Science<br>Indexes searched: SCI-EXPANDED, SSCI, ESCI<br>Fields searched: Topic (Title, Abstract, Author Keywords, Keywords Plus)<br>Date searched: 1 January 2026<br>Timespan: 2014–2026<br>Indexes: SCI-EXPANDED, SSCI, ESCI	1. TS= ("Clinical pharmacist")<br>2. TS= ("Community pharmacist")<br>3. TS= ("Pharmaceutical care")<br>4. TS= ("Medication therapy management")<br>5. TS=("Pharmacovigilance")<br>6. TS= ("Medication review")<br>7. TS= ("Therapeutic drug monitoring")<br>8. TS= ("Clinical pharmacy")<br>9. TS= ("Community pharmacy")<br>10. 1 or 2 or 3 or 4 or 5 or 6 or 7 or 8 or 9<br>11. TS= ("Romania")<br>12. 10 and 11<br>13. Refined by publication years: 2014–2026
Search platform: Elsevier Scopus<br>Fields searched: TITLE-ABS-KEY<br>Date searched: 1 January 2026<br>Document types included:<br>• Article<br>• Review (if containing original Romanian data relevant to pharmacist role)	1. TITLE-ABS-KEY ("Clinical pharmacist")<br>2. TITLE-ABS-KEY ("Community pharmacist")<br>3. TITLE-ABS-KEY ("Pharmaceutical care")<br>4. TITLE-ABS-KEY ("Medication therapy management")<br>5. TITLE-ABS-KEY("Pharmacovigilance")<br>6. TITLE-ABS-KEY ("Medication review")<br>7. TITLE-ABS-KEY ("Therapeutic drug monitoring")

	8. TITLE-ABS-KEY ("Clinical pharmacy")<br>9. TITLE-ABS-KEY ("Community pharmacy")<br>10. 1 or 2 or 3 or 4 or 5 or 6 or 7 or 8 or 9<br>11. TITLE-ABS-KEY ("Romania")<br>12. 10 and 11<br>13. 12 and PUBYEAR > 2013<br>14. 13 and PUBYEAR < 2027<br>15. Limit to document type: Article<br>16. Limit to document type: Review (if containing original Romanian data)
